# Enhancing food security while reducing environmental impacts: Life cycle assessment of cultivation-irrigation systems and yield gap closure in paddy fields

**DOI:** 10.1016/j.heliyon.2025.e42028

**Published:** 2025-01-16

**Authors:** Abdullah Darzi-Naftchali, Markus Berger, Fereshteh Batoukhteh, Ali Motevali

**Affiliations:** a Water Engineering Department, Sari Agricultural Sciences and Natural Resources University, Sari, Iran; b Multidisciplinary Water Management Group, Faculty of Engineering Technology, University of Twente, Enschede, Netherlands; c Department of Mechanics of Biosystem Engineering, Sari Agricultural Sciences and Natural Resources University, Sari, Iran

**Keywords:** Dry-direct seeding, Drip irrigation, Life cycle assessment, Water productivity, Yield

## Abstract

Enhancing food and water security is crucial, not only by preventing additional burdens on the environment but also by significantly mitigating existing environmental challenges. Dry direct-seeding of rice (DDSR) and yield gap (YG) reduction are effective in enhancing water productivity (WP) and food production. However, their related environmental impacts (EI) have received less attention. This study uses life cycle assessment and farmers' field data to analyze the EI of five rice cultivation-irrigation systems: transplanting with continuous flooding (T-CF), transplanting with alternate wetting and drying (T-AWD), DDSR with drip irrigation (DDSR-D), DDSR with sprinkler irrigation (DDSR-S), and DDSR with furrow irrigation (DDSR-F). Additionally, the energy productivity (EP) and WP of these systems were assessed. An environmental efficiency of YG reduction (EE) index was introduced as the ratio of EI reduction to YG reduction to evaluate the EI of closing YG. DDSR-D significantly reduced water, electricity, diesel fuel, and machinery use by 61–200 %, 14–64 %, 7–98 %, and 13–46 %, respectively. DDSR-D achieved the highest rice yield, which was 3.8 %, 4.4 %, 18.4 %, and 7.7 % higher than T-CF, T-AWD, DDSR-S, and DDSR-F, respectively. Compared with transplanting, DDSR increased EP and WP by 19.2 % and 63.8 %, respectively, and reduced CH_4_ and CO_2_ emissions by 26.5 % and 95.4 %, respectively. Overall, DDSR-D decreased the total EI by approximately 36.3 %, 13.3 %, 26.7 %, and 3.1 % compared to T-CF, T-AWD, DDSR-S, and DDSR-F, respectively. Sensitivity analysis indicated that EI was most impacted by electricity and diesel fuel consumption. Increasing the minimum yield by 80 %, 143 %, 205 %, 260 %, 310 %, and 322 % resulted in EI reductions of 77.8 %, 115.4 %, 159.2 %, 195.5 %, 209.6 %, and 193.4 %, respectively. The EE index was 0.36 Pt kg^−1^ for closing YG up to the average yield and 0.04 Pt kg^−1^ for closing YG from average to maximum yield, indicating that lower yields contribute more to environmental degradation. Based on the results, incorporating YG reduction strategies in DDSR-D can enhance the environmental sustainability of rice production and accelerate progress toward achieving sustainable development goals.

## Highlights

• Life cycle assessment evaluated environmental impact (EI) of dry direct-seeding rice (DDSR).
• DDSR with drip irrigation (DDSR-D) reduced CH_4_ (96.9 %) and CO_2_ (30.4 %) emissions.
• DDSR-D increased water (205 %) and energy (51.4 %) productivity while boosting yield (3.8–18.4 %).
• Closing the crop yield gap (YG) reduced EI by up to 210 %.
• The economically yield was identified as the optimal target for YG reduction concerning EI.

## 1 Introduction

The increased food insecurity since 2015 indicates the agricultural sector's failure to address Sustainable Development Goal 2 (SDG2), zero hunger, at its core [102]. While this sector accounts for over 71 % of the world's renewable water withdrawal [106], it also poses a serious threat to environmental sustainability through greenhouse gas (GHG) emissions, deforestation, land use change, acidification, eutrophication, and biodiversity loss [21,86]. Sufficient food production in the agricultural sector is vital for improving food availability and achieving food security. However, minimizing the sector's potentially harmful environmental impact (EI) is essential to meet sustainability criteria.

Rice (*Oryza sativa*), cultivated on about 165 million ha with a production of 778 million tons [26], plays a crucial role in the nutrition, income, and employment of millions worldwide [115]. However, rice production systems significantly impact the environment due to high water consumption, fertilizer losses, extensive pesticide use, and GHG emissions [103]. The conventional irrigation method for paddy fields in many regions is continuous flooding, which increases nutrient availability and helps control weeds [114]. However, the EI of paddy fields is substantial due to high water consumption and low productivity [65,107], leading to significant water loss, particularly through deep percolation [20].

Reducing the yield gap (YG: the difference between observed and attainable yield) of rice can be a viable solution to enhance food security and meet future rice demand. Surveys indicate varying values for the rice YG (up to 6.4 t ha^−1^) across different regions worldwide [80,89,90]. It has been reported that closing this YG and achieving 100 % of the attainable yield could increase global rice production by approximately 47 % [75]. Among the many factors effective in closing the rice YG, improving water management is particularly crucial given the current water scarcity situation. During the last decades, various efforts have been made to improve water use efficiency (WUE) in paddy fields. These efforts include saturated rice culture, mid-season drainage, aerobic rice cropping, intermittent flooding, alternate wetting and drying, the system of rice intensification, dry direct-seeding of rice (DDSR), furrow-irrigated rice, drip-irrigated rice, sprinkler-irrigated rice, automatic irrigation systems, and many other practices such as mulching, minimum or reduced tillage, and the use of stress-tolerant varieties.

The effectiveness of water-saving strategies and cropping systems in increasing WUE compared to conventional transplanting-continuous flooding (T-CF) culture has been confirmed in several studies. A review by Arouna et al. [6] indicated that water conservation technologies significantly improved WUE compared to T-CF. Another comprehensive review by Mallareddy et al. [65] confirmed that water-efficient technologies reduce water consumption and enhance the productivity of rice cultivation. Fukai and Mitchell [28] reviewed papers on aerobic rice culture and demonstrated that this culture improved WUE by more than 50 % compared to T-CF. This culture can reduce GHG emissions and improve grain quality by lowering the total arsenic and mercury content in rice grains, as soil drying hinders their solubilization and methylation in the rhizosphere, leading to reduced uptake [15,48]. Compared to T-CF, the water-saving strategies increased rice yield in some cases [9,23,37,54,61,109] and decreased it in other conditions [52,58].

Overall, the benefits of water-saving strategies have provided a strong incentive for policymakers and farmers to adopt these systems in rice fields. While the effects of conventional aerobic methods on crop production and water consumption have been extensively studied, and their EI has been estimated, the EI of DDSR has received very little attention. DDSR entails cultivating rice in dry, un-puddled fields by sowing seeds directly rather than transplanting seedlings [17,56,61]. This method is gaining popularity across various countries due to its potential benefits. While flooding irrigation is predominant in transplanting, DDSR also allows using drip, sprinkler, and furrow irrigation systems. Before implementing on a large scale, it is crucial to assess the performance of various irrigation systems in DDSR using comprehensive indices. Considering the diversity and extent of EI of various irrigation systems and strategies in paddy fields, the concept of life cycle assessment (LCA) can be a suitable tool to identify, quantify, and evaluate the resources consumed and the emissions from paddy rice cultivation [32].

LCA has increasingly been used to assess the EI of rice production across various farming practices and regions. Studies have examined the environmental footprint of different rice systems, including conventional, organic, and intensive practices, with a focus on GHG emissions, water use, and biodiversity [2,11,72,74,76,100,110]. Research has highlighted the potential for improved resource efficiency through methods like the System of Rice Intensification (SRI) and alternative cropping systems such as rice-cowpea rotations [29,83]. Findings suggest that adopting sustainable practices, such as integrated pest management and optimized water use, can reduce environmental pressures and improve eco-efficiency in rice farming [14,35]. These studies underscore the value of LCA in identifying opportunities for sustainability improvements and enhancing the environmental performance of rice production globally.

A few studies have utilized LCA to comprehensively investigate the combined environmental challenges posed by irrigation systems and water management practices in paddy fields. In related research, Darzi-Naftchali et al. [22] and Giuliana et al. [30] found that better aeration reduces environmental issues of rice production. In another recent study using LCA, Wang et al. [104] found that drip-irrigated DDSR reduced impacts on water resources and climate change compared with T-CF. The prior studies have overlooked the EI of DDSR under various irrigation systems and the EI of YG closure in rice production. To address this gap, this study aims to compare the EI of T-CF with and DDSR under different irrigation systems, evaluate crop yield, water productivity, and energy productivity in T-CF and DDSR, and assess the EI of closing the rice YG relative to DDSR impacts. A significant contribution of this research is its particular emphasis on the innovative technique of DDSR in conjunction with irrigation methods such as drip, sprinkler, and furrow irrigation as well as emphasis on YG reduction as a vital strategy for enhancing food security. By introducing the "environmental efficiency of yield gap reduction" index, this study establishes an innovative framework for assessing the trade-offs between increasing agricultural yields and minimizing environmental harm. This focus on YG reduction is essential for achieving SDG 2 (Zero Hunger), as closing the yield gap can significantly enhance rice production without necessitating additional agricultural land, thereby improving food availability in resource-limited areas. Furthermore, the analysis of water productivity and energy productivity illustrates how DDSR systems can enhance resource efficiency while addressing the pressing need for sustainable water management, thereby contributing to SDG 6 (Clean Water and Sanitation). The study's findings regarding reduced GHG emissions, along with decreases in fuel and energy consumption, underscore its relevance to SDG 13 (Climate Action) by advocating for climate-smart agricultural practices. In summary, by integrating YG reduction strategies with DDSR and advanced irrigation techniques, this research presents a novel and actionable approach to optimizing rice production. It not only tackles food security issues but also accounts for the environmental consequences of rice farming, making a significant contribution toward achieving key Sustainable Development Goals and promoting global sustainability.

## 2 Materials and methods

### 2.1 Study area

**Fig. 1:**
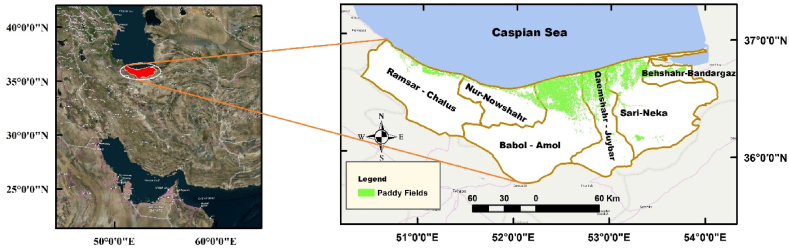
Geographical location of the study area in the north of Iran.

Mazandaran province, situated in the northern part of Iran along the southern coast of the Caspian Sea, was selected as the study area (Fig. 1). This province leads the nation in rice production, accounting for 39.6 % of the total rice cultivation area and 44 % of the total rice production [69]. Mazandaran province has a total annual renewable water supply of 6000 million cubic meters (MCM), comprising 4500 MCM of surface water and 1500 MCM of groundwater, with approximately 55 % of the surface water and 87 % of the groundwater being utilized. Agriculture is the primary water-consuming sector in the province, accounting for 88 % of the total water consumption, while the municipal and industrial sectors consume 10 % and 2 %, respectively [78]. In terms of water management, the province is divided into six independent plains: Ramsar-Chalus, Nur-Nowshahr, Babol-Amol, Qaemshahr-Juybar, Sari-Neka, and Behshahr-Bandargaz. Mazandaran's average annual rainfall, evaporation, and temperature are 637 mm, 434 mm, and 13.6 0C, respectively. The amount of rainfall decreases from the west to the east of the province, so the long-term average rainfall in these plains is 768, 661, 593, 631, 595, and 587 mm, respectively. In addition, recent years have seen a general decline in rainfall compared to the long-term average. In 2023, the province experienced a 20 % decrease in rainfall, while the specific plains of Ramsar-Chalus, Nur-Nowshahr, Babol-Amol, Qaemshahr-Juybar, Sari-Neka, and Behshahr-Bandargaz saw a 19 %, 3 %, 23 %, 21 %, 26 %, and 14 % reduction in rainfall compared to the long-term average. Most of the agricultural lands, especially irrigated paddy fields, are located in the central and eastern regions of the province. Therefore, a significant decrease in rainfall in these plains severely impacted the sustainability of water resources and agriculture. One of the critical indicators of increasing instability in these plains is the significant drop in groundwater levels. Compared to the long-term average, the groundwater reservoir deficits are 4.56, 21.09, 41.12, 7.34, 20.76, and 61.74 MCM for Ramsar-Chalus, Nur-Nowshahr, Babol-Amol, Qaemshahr-Juybar, Sari-Neka, and Behshahr-Bandargaz, respectively [77].

### 2.2 Data collection

#### 2.2.1 Cultivation and irrigation systems

Transplanting is the common method of rice cultivation in the paddy fields of Mazandaran province. However, in recent years, some farmers in the Sari-Neka and Behshahr-Bandargaz plains have adopted DDSR cultivation. In transplanting cultivation, the predominant irrigation method is continuous flooding (T-CF), with some farms also employing AWD (T-AWD). In the fields cultivated using DDSR, farmers utilize furrow (DDSR-F), drip (DDSR-D), and sprinkler (DDSR-S) irrigation systems. The paddy fields in the area were irrigated using groundwater. Given these conditions, at least three farmer fields cultivated Hashemi rice, the major rice cultivar grown in Mazandaran province, were selected in the Sari-Neka and Behshahr-Bandargaz plains and monitored throughout the rice growing season in 2023. To collect the required information in these fields, a questionnaire was designed based on the objectives of the study. Face-to-face interviews with selected farmers during frequent visits helped fill out the questionnaire. Key information recorded during field visits included plowing and sowing activities, water consumption, the number and duration of irrigation, the type and amount of energy used, and the type and quantity of other inputs such as fertilizers, insecticides, herbicides, fungicides, seeds, machinery, and labor.

#### 2.2.2 Yield gap scenarios

The YG data for transplanted rice cultivation in Mazandaran province was obtained from field surveys and Saberali and Darzi-Naftchali [90]. Their study involved a comprehensive assessment of factors influencing the YG by surveying 1698 farmer fields in 2019, conducting crop modeling, and performing experimental studies. In the present study, several YG scenarios were considered, including the minimum farmer yield (Ymin), average yield in farmers' fields (Ya), maximum recorded yield in farmers' fields (Ymax), and economic yield (Yeco). Yeco is considered as 80 % of potential yield (Ymax) of rice which is economically attainable. Three additional rice yield scenarios were included within the range of Ymin and Ymax, encompassing approximately 25 % lower (Ya1) and higher (Ya2) than Ya, as well as 50 % higher than Ya (Ya3). The Ymax was utilized as the benchmark to compare the impact of various scenarios. The YG scenarios were integral to comprehensively evaluating the EI of narrowing yield discrepancies in rice production. Some of the information on these YG scenarios is presented in Table 1.

**Table 1:** Information used for EI assessment of YG scenarios.

Scenario	Yield (kg ha^−1^)	Pesticides		N (kg ha^−1^)	P (kg ha^−1^)	K (kg ha^−1^)	Water management	Water use (m^3^ ha^−1^)
		(Kg ha^−1^)	(L ha^−1^)					
Ymin	1721	0	0	30	0	0	CF	8435
Ya1	3100	0	0.6	50	0	0	CF	9170
Ya	4180	0.8	0.4	60	50	0	CF	9035
Ya2	5250	0.8	1.5	80	50	30	CF	10114
Ya3	6200	2.5	0	75	70	50	AWD	8470
Yeco	7054	5.5	2.0	130	30	50	CF	9528
Ymax	7264	3.0	1.4	300	75	75	AWD	7835

### 2.3 LCA methodology

The LCA concept evaluates the potential EI of a product, process, or system throughout its entire lifespan. This involves gathering, compiling, and quantifying all inputs and outputs. LCA offers a comprehensive assessment of energy and material consumption over the life cycle. According to ISO 14044 [51], LCA comprises four main stages: Goal and Scope Definition, (b) Life Cycle Inventory Analysis (LCI), (c) Life Cycle Impact Assessment (LCIA), and (d) Life Cycle Interpretation of the results.

#### 2.3.1 Goal and scope definition

**Fig. 2:**
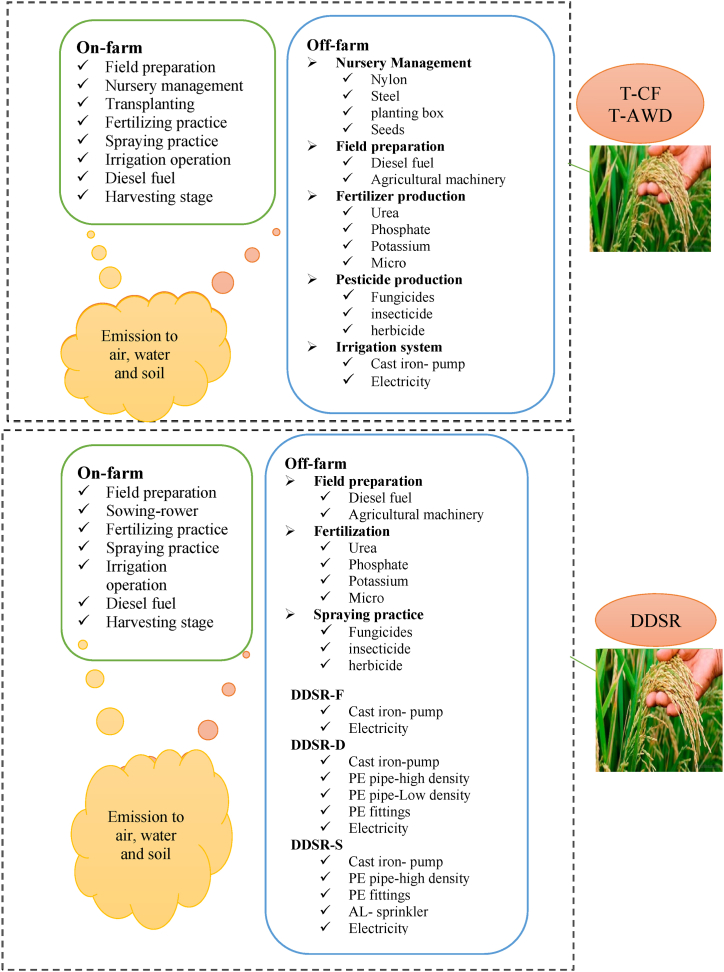
On-farm and off-farm data collected for LCA analysis.

The goal of this LCA was to evaluate the EI of different cultivation-irrigation systems and YG reduction scenarios in rice fields, offering insights to support decision-making for enhancing the sustainability of these agricultural practices. A cradle-to-gate approach was employed for this evaluation without applying any intentional cut-off criteria. The functional unit for all analyses was defined as one ton of unmilled rice grain, ensuring that all EI associated with rice cultivation were attributed to the rice grains. Consistent with similar studies [73], no allocation was made for rice straw as a co-product in paddy fields due to its negligible economic profit for farmers in the region. Consequently, the impacts of all activities ending with rice harvest, including the production and consumption of inputs during the most recent real field operations and the machinery used, were considered. Fig. 2 illustrates the collected data at the system boundary.

#### 2.3.2 Life Cycle Inventory Analysis

To perform the LCI, foreground and background datasets are utilized. The foreground data represents the study system, directly collected from the involved farms. This includes data on the application of pesticides, chemical fertilizers, fuel, and other resources, and the directly resulting emissions. The background LCI data encompasses the environmental interferences of producing fertilizers, fuels, electricity, irrigation system equipment, and other related inputs. This data is obtained from the Ecoinvent database in SimaPro 9.4.

The use of synthetic or manure fertilizers to promote crop growth and obtain higher gain yields results in several important emissions, including emissions of nitrous oxide (N_2_O), ammonia (NH_3_), and CO_2_ emissions (from urea) to air and nitrate (NO_3_-) emissions to water due to nitrogen-containing synthetic or organic fertilizer application, emissions of phosphorous-containing elements to water due to phosphorus in synthetic and manure fertilizers that affect global warming, eutrophication, and other impact categories. Based on previous studies, standard coefficients were selected to calculate the emissions caused by the consumption of fertilizers on farms [46]. The coefficients of emissions per unit of energy consumption in diesel-fuel burning processes based on the Ecoinvent database are presented in Table 2.

**Table 2:** The coefficients of emissions per unit of energy consumption in diesel-fuel burning processes.

Emissions	Coefficient (g/MJ)
Carbon dioxide (CO_2_)	74.5
Sulfur dioxide (SO_2_)	0.0241
Methane (CH_4_)	0.00308
Benzene (C_6_H_6_)	0.000174
Cadmium (Cd)	0.000000239
Chromium (Cr)	0.00000119
Copper (Cu)	0.0000406
Dinitrogen monoxide (N_2_O)	0.00286
Nickel (Ni)	0.00000167
Zink (Zn)	0.0000239
Benzo (a) pyrene	0.000000716
Ammonia (NH_3_)	0.000477
Selenium (Se)	0.000000239
PAH (polycyclic hydrocarbons)	0.0000785
Hydro carbons (HC, as NMVOC)	0.068
Nitrogen oxides (NOx)	1.06
Carbon monoxide (CO)	0.15
Particulates (b2.5 μm)	0.107

Heavy metals are added to the soil due to using of synthetic or manure fertilizers and due to deposition. The heavy metal content of fertilizers and manure was tabulated in Table 3. Emissions from the use of pesticides depend on its active ingredient. The percentage and type of active ingredients of the pesticides used were obtained from the manufacturing companies. It is estimated that 90 % of the active ingredient is released into the soil, while the remaining 10 % is released into the air [67].

**Table 3:** Heavy metal content of fertilizers.

Fertilizers	Unit	Heavy metal coefficient						
		Cd	Cu	Zn	Pb	Ni	Cr	Hg
N-fertilizer	mg kg^−1^ N	6	26	203	54.9	20.9	77.9	0.1
P-fertilizer	mg kg^−1^ P_2_O_5_	40	90.5	839	67	88.3	543	0.3
K-fertilizer	mg kg^−1^ K_2_O	0.1	4.8	6.2	0.8	2.5	5.8	0

#### 2.3.3 Life Cycle Impact Assessment and Interpretation

Simapro 9.4 and the ReCiPe2016 method were used to evaluate EI. ReCiPe2016 provides a state-of-the-art method to classify life cycle assessment results through 17 groups of intermediate effects (midpoint indicators) and three groups of endpoint effects on human health, natural environment, and resource scarcity [42,43]. For human health, the method combines indicators such as particulate matter, tropospheric ozone formation, ionizing radiation, stratospheric ozone depletion, human toxicity (cancer and non-cancer effects), global warming, and water use to assess impacts on human well-being. For ecosystem quality, it includes global warming, water use, freshwater toxicity and eutrophication, tropospheric ozone, terrestrial ecotoxicity, terrestrial acidification, land use/transformation, and marine ecotoxicity, which together reflect the effects on biodiversity and ecosystem health. The resource availability endpoint evaluates the depletion of non-renewable resources like fossil fuels and minerals. By aggregating these three endpoints, the final EI provides a holistic measure of the overall environmental burden associated with different cultivation-irrigation systems and YG reduction scenarios across their entire life cycle. At the final stage in LCA, the environmental damage results of each scenario are interpreted by identifying significant parameters strongly influencing the results as well as a completeness, consistency, and sensitivity analysis of these parameters. The sensitivity analysis was done by adjusting the quantities of N fertilizer, water, electricity, and diesel-the most influential input parameters-by ±25 % and then comparing the resulting changes in EI to the baseline.

### 2.4 Evaluation indices

Three additional indices including energy productivity (EP), water productivity (WP), and environmental efficiency of YG reduction (EE) index were also used to assess the effectiveness of different cultivation and irrigation systems as well as YG closure scenarios. EP (kg MJ^−1^) was determined as the ratio of rice yield (kg ha^−1^) to the total energy input (MJ ha^−1^). The direct input energy from seeds, diesel fuel, human labor, chemical fertilizers, biocides, and irrigation water use was calculated by multiplying the energy equivalent (Table 4) by the amount of their use in one ha. The indirect input energy used in agricultural machinery was computed from the total weight, useful life, energy coefficient, and operating time according to Equation 1 [96].

**Table 4:** The energy equivalent of consumption inputs.

Input	Unit	Energy equivalent (MJ unit^−1^)	References
Human labor	hr	1.96	[39]
**Machinery**			
Tractor	kg	93.61	[82]
Combine	kg	87.63	[82]
Plow	kg	62.7	[82]
Disc harrows	kg	62.7	[82]
Chisel	kg	62.7	[82]
Furrower	kg	62.7	[82]
Rower	kg	62.7	[82]
Sprayer	kg	62.7	[82]
Rotary tiller	kg	62.7	[55]
Transplanter	kg	51.5	[91]
Diesel fuel	l	56.31	[39]
Chemical fertilizers			
Nitrogen (N)	kg	66.14	[39]
Phosphate (P_2_O_5_)	kg	12.44	[39]
Potassium (K_2_O)	kg	11.15	[39]
Micro	kg	120	[39]
Animal Manure	kg	23.5	[55]
Pesticides	l	102	[40]
Pesticides	kg	120	[40]
Water for irrigation	m^3^	1.02	[70]
Electricity	kwh	11.93	[39]
Seeds (Rice)	kg	14.7	[57]
Irrigation system			
Pump	kg	142.7	[55]
PE pipe	kg	91.65	[44,45]
PE fittings	kg	98.5	[44,45]
Sprinkler-AL	kg	181.06	[92]
Nylon	kg	158	[4]

ME = $\frac{W}{L\;X\;A}$ x ECF x T (1)

where ME is agri-machinery energy (MJ ha^−1^), ECF is the energy conversion factor for agri-machinery used (MJ kg^−1^), W is the weight of agri-machinery (kg), L is the useful life of the agri-machinery (hr), T is the working time (hr), and A is the size of the farm (ha). The total amount of energy input is determined by adding all energy equivalents.

Global Warming Potential (GWP) is obtained from the output of the Simapro model as the amount of additional radiative forcing caused by an emission of 1 kg of GHG relative to the additional radiative forcing caused by the release of 1 kg of CO_2_ [43]. WP (kg m^−3^) was calculated as the ratio of crop yield (kg ha^−1^) to water consumption (m^3^ ha^−1^) [25]. EE (Pt kg^−1^) was introduced in this research as the ratio of EI reduction (Pt ha^−1^) to YG reduction (kg ha^−1^). In order to express EE in one number only, the weighted ReCiPe 2016 (HI) single-score result has been used to determine EI.

### 2.5 Statistical analysis

Statistical analyses were performed using SAS software (version 9.0) to evaluate the effects of different cultivation-irrigation on resource use, emissions, crop yield, WP, and EP. The experiment was designed as a completely randomized design with three replications for each treatment (T-CF, T-AWD, DDSR-F, DDSR-D, DDSR-S). Data analysis was conducted using the General Linear Model (GLM) procedure in SAS, with each environmental parameter (input data and emissions to air, water, and soil) specified as the dependent variable and the treatment as the independent variable. The model was fitted using Type III sum of squares (SS3) to account for the unbalanced design. Post-hoc mean comparisons were carried out using the least significant difference (LSD) method to identify significant differences between treatments at a significance level of P ≤ 0.05.

## 3 Results

### 3.1 Resource consumption

The major input used in different treatments for one-ton paddy rice production is presented in Table 5. There was a significant difference between machinery in T-CF and DDSR-D. Macro and micronutrient consumption was not significantly different in various treatments. However, consumption of urea, phosphate, and potassium in the DDSR treatments was 6.1 %, 7.9 %, and 23.6 % more than those in transplanting, respectively. The maximum pesticide consumption was related to DDSR-F, which was significantly higher than the corresponding value in T-AWD. Pesticides consumption in DDSR was 51.7 % higher than transplanting. Water consumption in T-CF was significantly higher than T-AWD (41.7 %), DDSR-D (66.7 %), DDSR-S (46.4 %), and DDSR-F (43.1 %). Electricity in T-CF was significantly (38.9 %) more than DDSR-D but there were no differences among T-CF, T-AWD, DDSR-S, and DDSR-F. Diesel fuel consumption in T-CF and T-AWD was significantly more than that in the DDSR treatments. On average, the DDSR treatments used 39.5 %, 11.5 %, and 45.8 % lower water, electricity, and diesel fuel for rice production compared to transplanting. Total input in the study area was in the order of T-CF > DDSR-S > T-AWD > DDSR-F > DDSR-D.

**Table 5:** Mean comparison of major input consumption for producing one-ton paddy rice in different cultivation and irrigation systems. Means followed by different letters are significantly different at P < 0.05.

Input	Treatment				
	T-CF	T-AWD	DDSR-D	DDSR-S	DDSR-F
Machinery (kg)	2.60^a^	2.15^ab^	1.78^b^	2.02^ab^	2.24^ab^
Urea (kg)	60.1^a^	64.4^a^	64.9^a^	65.0^a^	68.2^a^
Phosphate (kg)	22.2^a^	21.5^a^	21.9^a^	26.0^a^	22.8^a^
Potassium (kg)	22.2^a^	15.3^a^	21.7^a^	26.0^a^	21.8^a^
Micro (kg)	0.8^a^	1.0^a^	1.1^a^	0.7^a^	0.9^a^
Pesticides (L)	0.58^ab^	0.4^b^	0.56^ab^	0.69^ab^	0.98^a^
Water (m^3^)	2625^a^	1531^b^	874^c^	1406^b^	1443^b^
Electricity (KWh)	1544^a^	1099^ab^	944^b^	1483^a^	1079^ab^
Diesel fuel (L)	24.7^a^	24.9^a^	12.6^b^	14.2^b^	13.5^b^

### 3.2 Environmental impact

#### 3.2.1 Cultivation and irrigation systems

Results LCI.

The results of the mean comparison of the emissions to air, water, and soil are presented in Table 6. Emissions to air in the transplanting treatments were significantly more than those in the DDSR treatments for all elements unless N_2_O and pesticide. The trend was not observed for the emissions to soil and water. Generally, the maximum emission of different elements into soil and water occurred in DDSR-S. The DDSR treatments reduced CO_2_ and CH_4_ emissions to air by 26.5 % and 95.4 %, respectively. Compared with T-CF, CO_2_ emission to air slightly increased in T-AWD (2.6 %) but significantly decreased in DDSR-D (30.4 %), DDSR-S (20.1 %), and DDSR-F (26.3 %). Under T-CF, the emission of CH_4_ to air was 49.9 %, 96.9 %, 96.1 %, and 96.7 % more than that in T-AWD, DDSR-D, DDSR-S, and DDSR-F. The aerobic cultivation systems showed different responses in viewpoints of the emission of N_2_O to air and NO_3_- to soil. The T-AWD and DDSR-D slightly decreased these emissions while DDSR-S increased them by 16.7 % and 18.7 %, respectively. Pesticide emission to soil decreased by 43.3 %, 46.7 %, 46.7 %, and 50.0 % in T-AWD, DDSR-D, DDSR-S, and DDSR-F, respectively, compared to T-CF.

**Table 6:** On-farm emissions for producing one ton of paddy rice. Means followed by different letters are significantly different at P < 0.05.

Emissions	Treatments				
	T-CF	T-AWD	DDSR-D	DDSR-S	DDSR-F
**1. To air (kg)**					
CO_2_	145.5^a^	149.3^a^	101.2^b^	116.3^b^	107.3^b^
SO_2_	0.03^a^	0.03^a^	0.02^b^	0.02^b^	0.02^b^
CH_4_	54.1^a^	27.1^b^	1.7^c^	2.1^c^	1.8^c^
C_6_H_6_	2.5E-04^a^	2.3E-04^a^	1.0E-04^b^	1.0E-04^b^	1.0E-04^b^
Cd	3.1E-07^a^	3.3E-07^a^	1.7E-07^b^	1.9E-07^b^	1.8E-07^b^
Cr	1.6E-06^a^	1.7E-06^a^	8.5E-07^b^	9.5E-07^b^	9.1E-07^b^
Cu	5.3E-05^a^	5.6E-05^a^	2.9E-05^b^	3.2E-05^b^	3.1E-05^b^
N_2_O	0.48^b^	0.46^b^	0.47^b^	0.56^a^	0.50^ab^
Ni	2.2E-06^a^	2.3E-06^a^	1.2E-06^b^	1.3E-06^b^	1.3E-06^b^
Zn	3.1E-05^a^	3.3E-05^a^	1.7E-05^b^	1.9E-05^b^	1.8E-05^b^
Benzo (a) pyrene	9.4E-07^a^	9.9E-07^a^	5.1E-07^b^	5.7E-07^b^	5.5E-07^b^
NH_3_	1.50^a^	1.58^a^	0.87^b^	0.97^b^	0.93^b^
Se	3.1E-07^a^	3.2E-07^a^	1.7E-07^b^	1.9E-07^b^	1.8E-07^b^
PAH	1.0E-04^a^	1.1E-04^a^	5.6E-05^b^	6.2E-05^b^	5.9E-05^b^
HC, as NMVOC	0.089^a^	0.094^a^	0.049^b^	0.054^b^	0.052^b^
Nox	1.39^a^	1.47^a^	0.76^b^	0.84^b^	0.81^b^
CO	0.20^a^	0.21^a^	0.11^b^	0.12^b^	0.12^b^
Particulates	0.14^a^	0.15^a^	0.08^b^	0.09^b^	0.08^b^
Pesticide	0.035^a^	0.020^b^	0.018^b^	0.019^b^	0.018^b^
**2. To water (kg)**					
NO_3_	13.4^b^	12.7^b^	13.3^b^	15.9^a^	13.9^ab^
PO_4_	0.079^b^	0.072^b^	0.079^b^	0.094^a^	0.082^ab^
**3. To soil (kg)**					
Cd	1.26E-03^b^	1.2E-03^b^	1.25E-03^b^	1.5E-03^a^	1.3E-03^ab^
Cu	3.8E-03^b^	3.6E-03^b^	3.8E-03^b^	4.5E-03^a^	3.9E-03^ab^
Zn	0.032^b^	0.030^b^	0.032^b^	0.038^a^	0.033^ab^
Pb	0.005^b^	0.005^b^	0.005^b^	0.006^a^	0.0053^ab^
Ni	0.003^a^	0.003^a^	0.003^b^	0.004^a^	0.0034^ab^
Cr	0.017^b^	0.016^b^	0.017^b^	0.020^a^	0.018^ab^
Hg	1.0E-05^b^	12E-05^a^	13E-05^a^	7E-05^b^	14E-05^a^
Pesticide	0.30^a^	0.17^a^	0.16^a^	0.16^a^	0.15^a^

### 3.3 Results LCIA

**Fig. 3:**
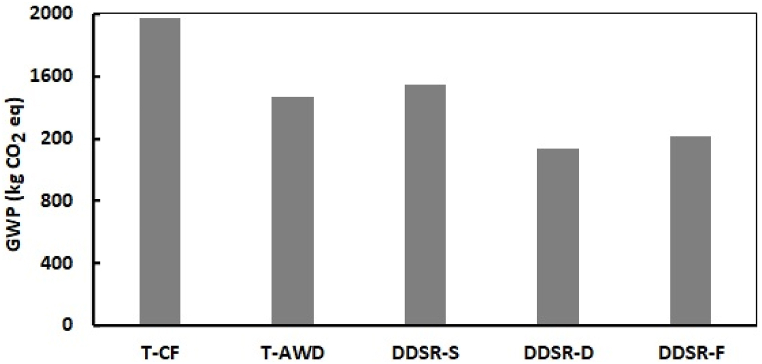
Global warming potential (GWP) in various cultivation-irrigation systems.

The influence of different cultivation-irrigation systems on global warming potential (GWP) is presented in Fig. 3. GWP values ranged from 1131 kg CO₂ eq for DDSR-D to 1975 kg CO₂ eq for T-CF. The considerable reduction in GWP in T-AWD (25.6 %) compared with T-CF suggests the high impact of alternate irrigation on GWP reduction. The effect was boosted by changing the cultivation method so that the amount of GWP in DDSR-F was 38.7 % less than that of T-CF. Drip irrigation had the greatest effect on reducing GWP. The GW reduction in DDSR-D compared to T-CF, T-AWD, DDSR-S, and DDSR-F was 74.6 %, 30.0 %, 36.9 %, and 7.0 %, respectively.

**Fig. 4:**
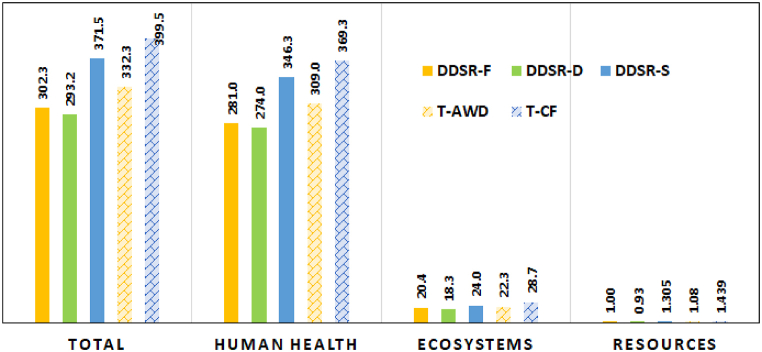
The total EI of cultivation-irrigation systems (Pt).

The impact of irrigation systems and cultivation methods on the total impact, human health, ecosystems, and resources is presented in Fig. 4. The minimum impact was related to DDSR-D followed by DDSR-F, T-AWD, DDSR-S, and T-CF, respectively. In transplanting cultivation, changing CF to AWD reduced the EI by about 16.8 %. In DDSR, the total impact in DDSR-D was 3 % and 21.1 % less than in DDSR-F and DDSR-S, respectively. In general, DDSR reduced the total impact, human health, ecosystems, and resources by about 11.9 %, 11.4 %, 18.1 %, and 14.4 % compared with transplanting treatments, respectively. Among the three endpoint impacts, the maximum impacts in all treatments occurred on human health, followed by ecosystems and resources.

**Figure 5:**
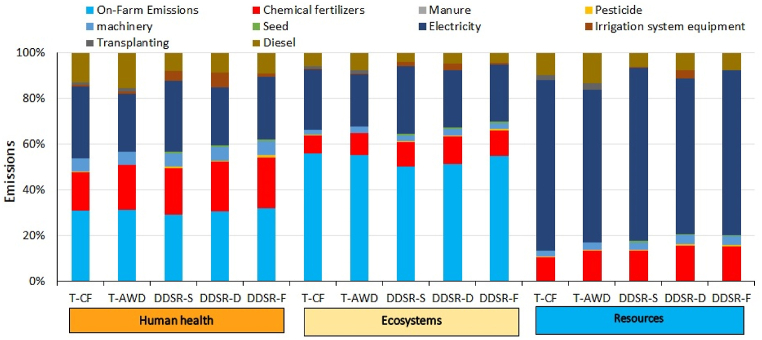
Contribution of processes to the three damage categories for rice production under different cultivation-irrigation systems.

The contribution of different factors to human health, ecosystems, and resources is presented in Fig. 5. In general, human health and ecosystems were influenced by 5 factors; in the order of on-farm emissions > electricity > chemical fertilizer > diesel > machinery. The total contribution of these 5 factors in human health was 97.5 %, 97.5 %, 94.1 %, 92.0 %, and 97.0 % in T-CF, T-AWD, DDSR-S, DDSR-D, and DDSR-F, respectively. This contribution in ecosystems was 98.6 %, 98.4 %, 97.1 %, 95.8 %, and 98.4 % in different treatments, respectively. The total contribution of pesticide, seed, irrigation system, and transplanting in human health and ecosystems in different treatments was in the range of 2.5%–8.0 % and 1.4%–4.2 %, respectively. In different treatments, about 66.8%–75.9 % and 6.3%–13.4 % of the negative effects on resources were related to electricity and diesel, respectively. The contribution of other factors in resources was in the range of 4.1%–8.0 %. The minimum and maximum impact of the irrigation system was related to T-CF and DDSR-D, respectively. The impact of irrigation systems on human health, ecosystems, and resources ranged from 0.7% to 6.4 %, 0.3%–3.0 %, and 0.0–3.6 % respectively.

### 3.4 Completeness, consistency, and sensitivity analysis

**Fig. 6:**
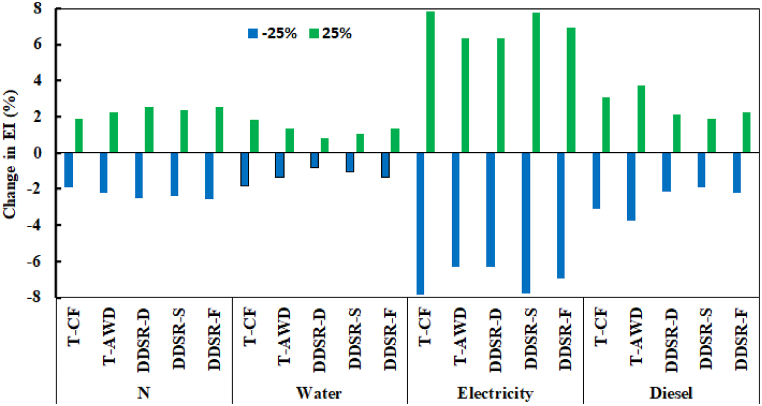
Impacts of ±25 % change in N fertilizer, water, electricity, and diesel on EI change compared to the baseline in different cultivation-irrigation systems.

As outlined in the goal and scope definition in Section 2.3, all parameters influencing rice production in each cultivation-irrigation system were comprehensively considered for the EI assessment. The primary inputs for rice production, which constitute the major part of the required data for the Simapro model, were directly collected from the study fields. These inputs included seeds, irrigation equipment, water, fertilizer, diesel, electricity, machinery, labor, and pesticides. A significance analysis revealed that the use of fertilizer, water, electricity, and diesel are the significant parameters influencing the results strongly. Based on a completeness and consistency check it can be stated that the data for these parameters is completely available and fulfills the requirements defined in the goal and scope definition. The results of the sensitivity analysis (Fig. 6) showed that EI was most sensitive to the change in electricity consumption (±7.8 %), followed by diesel fuel (±3.8 %), N fertilizer (±2.5 %), and water (±1.9 %). In various cultivation-irrigation systems, EI demonstrated differing sensitivities to various parameters. In DDSR, the sensitivity of EI to N fertilizer was higher compared to transplanting systems, while its sensitivity to water and diesel fuel was lower. For electricity consumption, the highest sensitivity was observed in T-CF and DDSR-S, with the lowest sensitivity found in DDSR-D.

#### 3.4.1 Yield gap closure scenarios

**Fig. 7:**
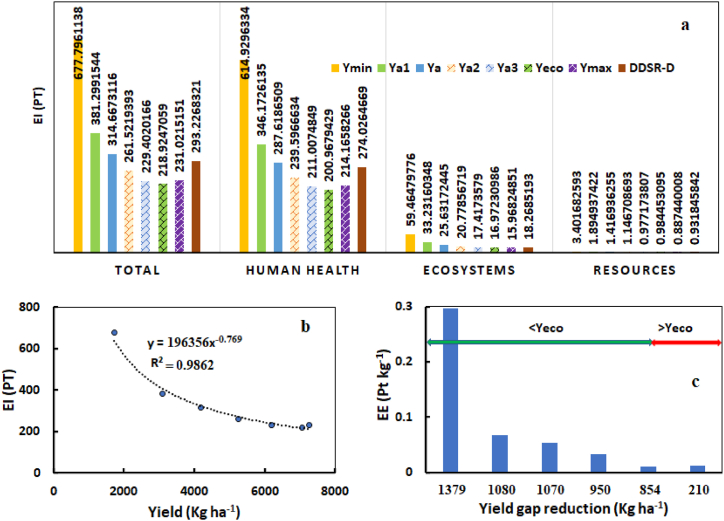
**The total EI in YG scenarios (a), the relationship between yield and EI (b), and the effect of YG reduction on EE (c)**.

The EI of YG closure scenarios and the best DDSR treatment as well as the relationship between yield and EI, and the effect of YG reduction and EE is presented in Fig. 7. The lowest yield had the highest EI, while the economic yield had the lowest impact. Increases in rice yield by 80 %, 143 %, 205 %, 260 %, 310 %, and 322 % resulted in reductions in EI by 77.8 %, 115.4 %, 159.2 %, 195.5 %, 209.6 %, and 193.4 %, respectively. The maximum observed yield was approximately 3 % higher than the economic yield, while its EI was about 5.5 % greater. The EI of Ymin, Ya1, Ya, Ya2, Ya3, Yeco, and Ymax were 0.68, 0.38, 0.31, 0.26, 0.23, 0.21, and 0.23 Pt kg^−1^, respectively. This impact was 0.29 kg Pt^−1^ for DDSR-D. The YG reduction was more effective in reducing EI for yields lower than Ya. The EE index was 0.36 Pt kg^−1^ and 0.04 kg Pt^−1^ for closing the YG from Ymin to Ya and from Ya to Yeco, respectively. This index in DDSR-D was 7.3 % lower than the corresponding one for the average yield in farmers' fields and 10.8 %, 21.8 %, 25.3 %, and 21.2 % higher than Ya2, Ya3, Yeco, and Ymax, respectively.

### 3.5 Crop yield and productivity of water and energy

**Fig. 8:**
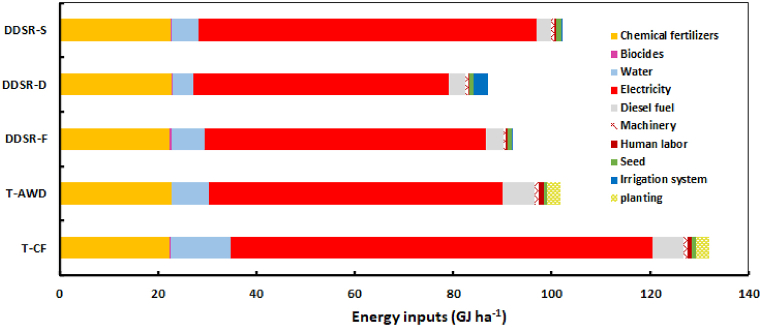
The share of energy inputs (GJ ha^−1^) for rice production under different irrigation-cultivation systems.

The contribution of different energy inputs for rice production is presented in Fig. 8. The maximum energy consumption was attributed to T-CF (132.1 GJ ha^−1^), followed by DDSR-S (102.2 GJ ha^−1^), T-AWD (101.9 GJ ha^−1^), DDSR-F (91.9 GJ ha^−1^), and DDSR-D (87.1 GJ ha^−1^). The total energy consumption in DDSR treatments was 20 % less than in transplanting treatments. DDSR-D reduced energy consumption by about 34.1 %, 14.5 %, 5.2 %, and 14.8 % compared to T-CF, T-AWD, DDSR-F, and DDSR-S, respectively. Electricity had a considerably higher contribution than the other energy sources, consisting of 65 %, 58.6 %, 62.3 %, 59.8 %, and 67.2 % of energy consumption in T-CF, T-AWD, DDSR-F, DDSR-D, and DDSR-S, respectively. Following electricity, chemical fertilizer, diesel fuel, and water contributed more than planting operations, human labor, machinery, seeds, pesticides, and irrigation equipment. Energy contributions from electricity, chemical fertilizer, diesel fuel, and water in the DDSR treatments were 18.5 %, 12.4 %, 49.4 %, and 44.4 % lower than in the transplanting treatments. Under transplanting conditions, a change in flooding irrigation to AWD decreased energy consumption by 22.9 %. In addition, a comparison of surface irrigation treatments indicated that the alternate irrigation in DDSR-F reduced energy consumption by about 9.8 % and 30.4 % compared with T-AWD and T-CF, respectively. The contribution of water in T-CF energy consumption was 38.0 %, 45.2 %, 65.8 %, and 53.8 % more than the corresponding one in T-AWD, DDSR-F, DDSR-D, and DDSR-S, respectively.

**Fig. 9:**
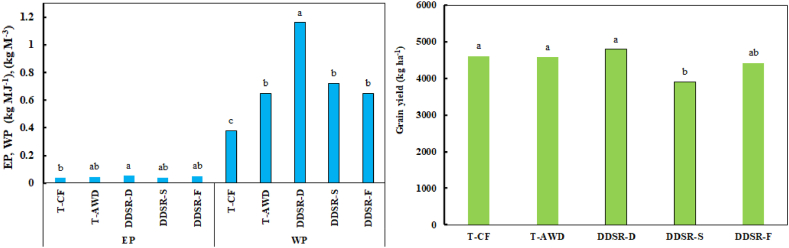
Influence of different cultivation-irrigation systems on EP, WP, and crop yield. Means followed by different letters are significantly different at P < 0.05.

The results of the mean comparison of the effect of different irrigation-cultivation systems on crop yield, EP, and WP are presented in Fig. 9. There were no significant differences among crop yields in T-CF, T-AWD, DDSR-D, and DDSR-F. Only in DDSR-S, the yield was significantly lower than the transplanting and DDSR-D treatments. In the highest-yielding treatment (DDSR-D), rice yield was 3.8 %, 4.4 %, 18.4 %, and 7.7 % more than T-CF, T-AWD, DDSR-S, and DDSR-F, respectively. There was only a significant difference between EP in DDSR-D and T-CF. DDSR-D resulted in a 34.0 %, 15.1 %, 24.7 %, and 5.7 % increase in EP compared with T-CF, T-AWD, DDSR-S, and DDSR-F, respectively. Also, DDSR-D improved WP significantly by 67.2 %, 44.0 %, 37.9 %, 44.0 compared with T-CF, T-AWD, DDSR-S, and DDSR-F, respectively. Compared to transplanting, on average, the DDSR treatments increased EP and WP by 19.2 % and 63.8 %, respectively.

## 4 Discussion

### 4.1 Resource consumption

Decades of efforts to reduce water consumption in worldwide rice production systems led to the introduction of the DDSR cultivation method under water-saving irrigation. The LCA assessment of the cropping system based on the farmer-field operations revealed the important role of water management and irrigation systems in environmental sustainability. Among various treatments, DDSR-D was the most input-efficient treatment to produce one-ton paddy rice in terms of machinery, water, electricity, and diesel fuel consumption. The higher nutrient consumption (without a significant difference) in DDSR compared to TP is partially due to the lack of appropriate technical recommendations for fertilizer management for such a cultivation system. In addition, since these farmers were used to consuming part of the fertilizer at transplanting in the conventional cropping system, they used the same method of fertilization in DDSR. In such a situation, a large part of consumed urea is lost through various processes [8,60]. In other words, only the cultivation and irrigation system has changed in DDSR. Therefore, farmers should be encouraged to adopt farm management suitable for DDSR. The development of pilot farms and extension services can improve the education level of farmers and enhance their acceptability [113].

The consumption of pesticides was significantly lower in transplanting than in DDSR due to the inhibitory role of permanent or periodic flooding of the field in weed growth [50,94]. Farmers tend to apply more herbicides to avoid rice yield loss due to weed competition, which can be as high as 66 % under aerobic conditions [27]. Intermittent wetting and drying of the soil, especially in the early stages of the growing season, makes it more difficult to control weeds in DDSR. Rao et al. [88] introduced minimizing weed seed production and dispersal, promoting seed predation and decay, preventing weed germination and emergence, and developing anaerobic germination as preventive approaches for weed control in DDSR. The evidence in the region showed that some farmers significantly reduced the growth rate of weeds by plowing-irrigation-plowing (after weeds have sprouted) operation. This operation increases machinery use and fuel, water, and energy consumption to some extent. Therefore, an appropriate measure should be chosen based on a comprehensive trade-off analysis.

Due to the elimination of deep plowing and puddling operations, DDSR resulted in lower machinery operation and diesel fuel consumption compared with transplanting. Moreover, reducing irrigation water consumption reduced pumping hours and consequently decreased the electricity consumption in DDSR. Improving the efficiency of machinery, electricity, and diesel fuel use in DDSR was also proven in past research [41,108,111]. A reduction of up to 66 % of water application in DDSR compared to T-CF was very promising. No need to use water for plowing and puddling operations, supplying crop water requirement at the right time, eliminating or significantly reducing deep percolation and evaporation, and eliminating runoff, especially in drip and sprinkler irrigation, are the most important reasons for reducing water consumption in DDSR. In the traditional TP system, the water requirement for land preparation is reported 150–250 mm, and seepage plus deep percolation losses are reported 1–5 mm d^−1^ in heavy soils and 25–30 mm d^−1^ in light soils [13,31]. In addition, evaporation losses of 3–7 mm d^−1^ were reported by Sivapalan [97] in the TP cultivation. Considering the consequences of excessive water consumption for rice cultivation in the study area, such as significant groundwater level drop and depletion, land subsidence, environmental flow violation [36,71], the indiscriminate development of rice cultivation areas should be avoided so as not to neutralize the effect of water saving in DDSR on improving environmental sustainability.

### 4.2 Environmental impact assessment

#### 4.2.1 Cultivation and irrigation systems

The DDSR treatments significantly reduced greenhouse gas emissions compared with T-CF, except for N_2_O emission to air in DDSR-S and DDSR-F. The variation in N_2_O emissions across different irrigation treatments can be attributed to differences in soil properties, field management, fertilizer management, and irrigation practices in farmers' fields. N_2_O emissions may rise under AWD conditions, which elevate soil redox potential and promote nitrification, with re-flooding potentially enhancing emissions through denitrification [7]. However, previous research has shown an increase in N_2_O emissions under DDSR or AWD [16,24,64] or no significant difference [111]. Considering the warming potentials of CH_4_ and N_2_O, 28 and 273 times higher than that of CO_2_ [24], the increased N_2_O emissions in DDSR-S may offset its positive effects on global warming achieved by reducing CH_4_ and CO_2_ emissions. A significant reduction in CH4 emissions observed in the aerobic treatments in the present study is inconsistent with several studies [33,108]. Liu et al. [63] demonstrated that DDSR with alternate wetting and drying (DDSR-AWD) can reduce CH₄ emissions by up to 96.8 % compared to transplanting. Furthermore, a comprehensive review by Nikolaisen et al. [81] found that DDSR reduced CH₄ emissions by 28 % compared to transplanting, while AWD, saturated culture, and rainfed conditions reduced emissions by 38 %, 66 %, and 70 %, respectively. This reduction is related to the aerobic soil conditions that suppress methanotroph activity and the shallower root distribution in DDSR, which enhances CH_4_ oxidation [12,105]. Switching from CF to AWD in transplanting increased CO_2_ emissions slightly but reduced CH_4_ emissions by half. CH_4_ emissions peak during certain rice growth stages due to heightened metabolism, and flooding exacerbates these emissions [18,63]. By avoiding waterlogging during these periods through AWD, it is possible to reduce CH_4_ emissions significantly. Therefore, AWD proves more effective than CF in mitigating greenhouse gas emissions, particularly CH_4_, which has a higher impact on climate change than CO_2_, especially in regions where DDSR is unsuitable.

Reducing GHG emissions in DDSR significantly mitigated GW compared to transplanting, consistent with the findings of Wang et al. [104]. The total amount of CH_4_ and N₂O significantly contributing to GW [53] was 94 % lower in DDSR than in traditional transplanting. Reports indicate that dry direct-seeded rice (DDSR) reduced global warming by 76.2 % [101] and by up to 58.3 % [108], compared to transplanting. Among the treatments, DDSR-D had the most substantial impact, achieving a 96 % reduction in these GHGs compared to transplanting. Giuliana et al. [30] highlighted that irrigation is responsible for approximately 80 % of GW in paddy fields. Consequently, efforts to reduce water losses during the growing season and avoid puddling in DDSR resulted in significant reductions in GW compared to transplanting. Changing puddling to zero tillage decreased GW potential by 42 % [34]. Additionally, under aerobic conditions, there is improved oxygen diffusion into the soil, leading to reduced CH_4_ emissions [49]. AWD was reported to reduce GW potential by 39.6 % [5] and 77 % [24] compared to T-CF.

Emission of pollutants in DDSR ultimately led to a reduction in EI on human health, ecosystems, and resources compared to transplanting. It was shown that aeration can substantially decrease CH_4_ emissions, thereby diminishing the EI of rice production [1,116]. Uncertainty and sensitivity analyses, which involved adjusting input variables of continuous flooding, demonstrated that intermittent flooding reduces EI by approximately 40 % compared to CF [30]. In a recent study applying LCA to assess DDSR, Wang et al. [104] compared drip-irrigated DDSR with CF, showing that DDSR reduced impacts on water resources and climate change. Liao et al. [59] recommended combining reduced nitrogen fertilizer with other field practices to mitigate environmental issues while maintaining crop production in transplanted paddy fields. However, the present study found that DDSR-D generally leads paddy fields toward a low-emissions pathway, supporting the goals of the Paris Agreement and Sustainable Development Goals in mitigating global warming.

The average sensitivity of EI across all cultivation-irrigation systems was significantly higher for electricity, being 5.46, 3.04, and 2.68 times more sensitive compared to diesel, N fertilizer, and water, respectively. Therefore, if comprehensive measures to reduce various inputs are not feasible, prioritizing reductions in electricity consumption, followed by diesel, N fertilizer, and water, would be more effective in decreasing EI. Previous studies have shown that electricity and fuel energy account for a significant portion of the total energy use in Iranian rice fields, highlighting a considerable potential for energy conservation [38,79,85,99] and consequently reducing EI. High fuel consumption is primarily due to low fuel prices and outdated machinery, suggesting that using modern equipment could enhance fuel use efficiency. A sustainable solution to decrease reliance on traditional energy sources in agriculture is the adoption of renewable energy, which can replace electricity and fossil fuels, further reducing EI [112]. Electricity consumption in the study area is elevated due to groundwater pumping. In the transplanting rice system, WP is generally low (0.1–1.6 kg m^−3^; [68]), resulting in significant water loss. Improving WP through better water management practices, such as AWD and switching from transplanting to DDSR, can reduce water pumping and electricity consumption, thereby lowering EI. Additionally, improved water management enhances N use efficiency in rice fields [21], which is typically low, around 20–50 % [19]. Therefore, efforts to increase N use efficiency in rice production can significantly mitigate EI.

#### 4.2.2 Yield gap closure scenarios

The YG reduction demonstrated substantial potential to reduce the EI per unit of crop yield. A comprehensive evaluation of the full set of these effects using LCA has not been conducted until now. However, past research has emphasized the reduction of specific environmental effects, such as nitrogen and water losses, through YG reduction [75,98]. It is possible to significantly reduce the YG without significant cost increases by improving field practices and management of fertilizer, water, pesticides, and other inputs [90,93]. Analysis of the evaluation index shows a more significant positive effect of each unit of YG reduction on decreasing EI in the range of minimum to average yield rather than in the range of average to maximum yield. Among the various inputs, higher yields up to the economic yield can be achieved by using more fertilizers, especially N, and by controlling pests and diseases. As shown in Table 1, increasing the minimum yield to the average yield was achieved by increasing the consumption of N and P by 30 and 50 kg ha^−1^, respectively, and through better pest and disease control. Both crop yields were obtained under flooding irrigation, with average yields requiring only 7 % more water than the minimum yield. In other words, there are both economic and environmental incentives to address many factors contributing to the YG. The maximum yield observed on the farmer's field caused a slight increase in EI compared to the research farm, primarily due to a significant increase in fertilizer consumption. The maximum observed yield was about 3 % higher than the economic yield of the Hashemi rice cultivar in the region [90]. The analysis of EI indicates that exceeding the economic yield by increasing input consumption can diminish the effectiveness of YG reduction, both economically and environmentally. Therefore, a balanced fertilization program is crucial for optimal rice production [90] and to mitigate environmental issues such as high emissions to air, water, and soil, eutrophication of water bodies, and depletion of soil organic matter [66,87]. Closing the YG in the range from average to maximum yield in transplanting culture has reduced the EI of rice production to levels lower than those of the best DDSR method (i.e., DDSR-D). However, identifying and addressing factors contributing to the YG in DDSR, such as water management, nutrient application, crop rotation, plant density, weed control, disease management, and pest control, can enhance the environmental sustainability of this cultivation method [17].

### 4.3 Crop yield and productivity of water and energy

The better response of crop yield to DDSR-D was mainly due to improved resource use compared to the other treatments. Bhardwaj et al. [10] showed that DDSR with drip irrigation increased rice yield by about 15 % compared to T-CF. Aerobic rice cultivation improves soil conditions, boosts microbial activity, and enhances root growth, leading to higher rice yields and water conservation [3,62]. However, various studies have reported significant differences in rice yield between DDSR and CF, as well as among different irrigation techniques used in DDSR [33,47,54,61,104,108]. Singh et al. [95] reported a reduction in rice yield under DDSR-S compared to CF-T and DDSR-CF. Variations in rice yield across different regions are primarily due to differences in environmental and climate conditions, soil physicochemical attributes, field management practices, soil water tension, and crop cultivars [84]. Reduced water and energy consumption in aerobic cultivation caused a significant increase in WP (up to 1.16 kg ha^−1^) and EP (up to 0.053), especially in DDSR-D, providing more favorable condition for water savings. Increased WP in DDSR compared with CF observed in the present study is consistent with more efficient water consumption reported in some previous studies: 27–30 % increase [47], 89.1–93.6 % [10], 123.9 % [95], and 64.4–69.2 % [111]. Moreover, Hang et al. [33] reported that 11.4–88.9 % higher WP under water-saving strategies in DDSR than continuously flooded-DDSR. The positive influence of DDSR on EP was also demonstrated in different studies [108,111].

## 5 Conclusions

This study bridges significant gaps in existing literature regarding the environmental impacts (EI) of dry direct-seeding rice (DDSR) versus traditional transplanting under various irrigation systems, and the EI implications of closing the yield gaps (YG) in rice production. Our findings reveal that DDSR systems present a markedly lower EI compared to transplanting, with reductions in water use by 39.5 %, electricity by 11.6 %, diesel fuel by 45.8 %, and machinery by 15.2 %. Furthermore, total greenhouse gas emissions associated with DDSR were 70 % lower than those of transplanting, translating to a 33 % decrease in global warming potential. Importantly, the impacts of DDSR extend beyond direct resource use; it also reduced impacts on human health, the environment, and resources by 12.9 %, 22.1 %, and 16.8 %, respectively. The analysis also highlights the superior resilience of DDSR systems, evidenced by lower sensitivities to diesel, water, and electricity inputs compared to traditional methods. This indicates that DDSR is not only ecologically beneficial but also more efficient, yielding reduced EI even with varying input levels. The EI assessment of YG closure scenarios revealed a substantial reduction in environmental degradation potential, particularly through increasing yields to the average level. Enhancing the minimum yield to the average yield, the minimum yield to the economic yield, and the average yield to the economic yield decreased EI by 53.4 %, 67.7 %, and 16.3 %, respectively. The findings underscore that achieving higher yields through efficient input usage is imperative; striking a balance between yield optimization and resource consumption is essential for sustainable production. In conclusion, the research strongly advocates for incorporating DDSR and yield gap reduction strategies in rice production systems. These approaches not only promise more sustainable agricultural practices but also offer pathways to advance diverse Sustainable Development Goals (SDGs), including SDG 1 (No Poverty), SDG 2 (Zero Hunger), SDG 6 (Clean Water and Sanitation), and SDG 13 (Climate Action). Our findings encourage policymakers and practitioners to adopt DDSR techniques as a means to enhance productivity and sustainability, thus contributing to food security and environmental preservation.

## CRediT authorship contribution statement

**Abdullah Darzi-Naftchali:** Writing – review & editing, Writing – original draft, Software, Methodology, Funding acquisition, Formal analysis, Data curation, Conceptualization. **Markus Berger:** Writing – review & editing, Supervision, Methodology, Investigation, Conceptualization. **Fereshteh Batoukhteh:** Writing – original draft, Software, Investigation, Formal analysis. **Ali Motevali:** Writing – review & editing, Methodology, Investigation, Conceptualization.

## Ethics statement

This study was approved by the Ethics Committee of SANRU, with ethics approval reference 1403/66/71, dated October 07, 2024. The study involved adult farmers, and no participants were under the age of 18. Participants were informed about the aims of the study, their right to withdraw at any time, and how their data would be used. Confidentiality and anonymity of participants were strictly maintained. Obtaining written informed consent was not possible for this study. Instead, verbal consent was obtained from each participant, with clear and adequate reasoning for this approach explicitly.

## Data availability statement

Data will be made available on request.

## Funding

The authors would like to thank the 10.13039/100016920Sari Agricultural Sciences and Natural Resources University for supporting the project under contract number 02-1401-17.

## Declaration of competing interest

The authors declare that they have no known competing financial interests or personal relationships that could have appeared to influence the work reported in this paper.
